# Structure of poly[di­aqua­[μ-1,2-bis­(pyri­din-4-yl)ethane-κ^2^ *N*:*N*′]bis­(μ_3_-cyclo­butane-1,1-di­carboxyl­ato-κ^3^ *O*,*O*′:*O*′′:*O*′′′)dimanganese(II)]

**DOI:** 10.1107/S2056989015013791

**Published:** 2015-07-25

**Authors:** Do Nam Lee, Youngmee Kim

**Affiliations:** a Division of General Education (Chemistry), Kwangwoon Univeristy, Seoul 139-701, Republic of Korea; b Department of Chemistry and Nano Science, Ewha Womans University, Seoul 120-750, Republic of Korea

**Keywords:** crystal structure, α,ω-alkanedi­carboxyl­ate, manganese(II), cyclo­butane-1,1-di­carboxyl­ate (cbdc) ligand

## Abstract

In the title compound, [Mn(C_6_H_6_O_4_)(C_12_H_12_N_2_)(H_2_O)]_*n*_, the cyclo­butane-1,1-di­carboxyl­ate (cbdc) ligands bridge three Mn^II^ ions, forming layers parallel to the *ac* plane. These layers are additionally connected by 1,2-bis­(pyridin-4-yl)ethane ligands to form a three-dimensional polymeric framework. An inversion centre is located at the mid-point of the central C—C bond of the 1,2-bis­(pyridin-4-yl)ethane ligand. The coordination geometry of the Mn^II^ ion is distorted octa­hedral and is built up by four carboxyl­ate O atoms, one water O atom and a pyridyl N atom. The pyridine ligand and the coordinating water mol­ecule are in a *trans* configuration. One carboxyl­ate group of the cbdc ligand acts as a chelating ligand towards one Mn^II^ atom, whereas the second carboxyl­ate group coordinates two different Mn^II^ atoms.

## Related literature   

For rigid aromatic di­carboxyl­ate ligands for MOFs, see: Sumida *et al.* (2012 ▸). For flexible cyclo­hexa­nedi­carboxyl­ate ligands for MOFs, see: Lee *et al.* (2011 ▸); Kim *et al.* (2011 ▸). For flexible α,ω-alkanedi­carboxyl­ate ligands for MOFs, see: Hwang *et al.* (2012 ▸, 2013 ▸).
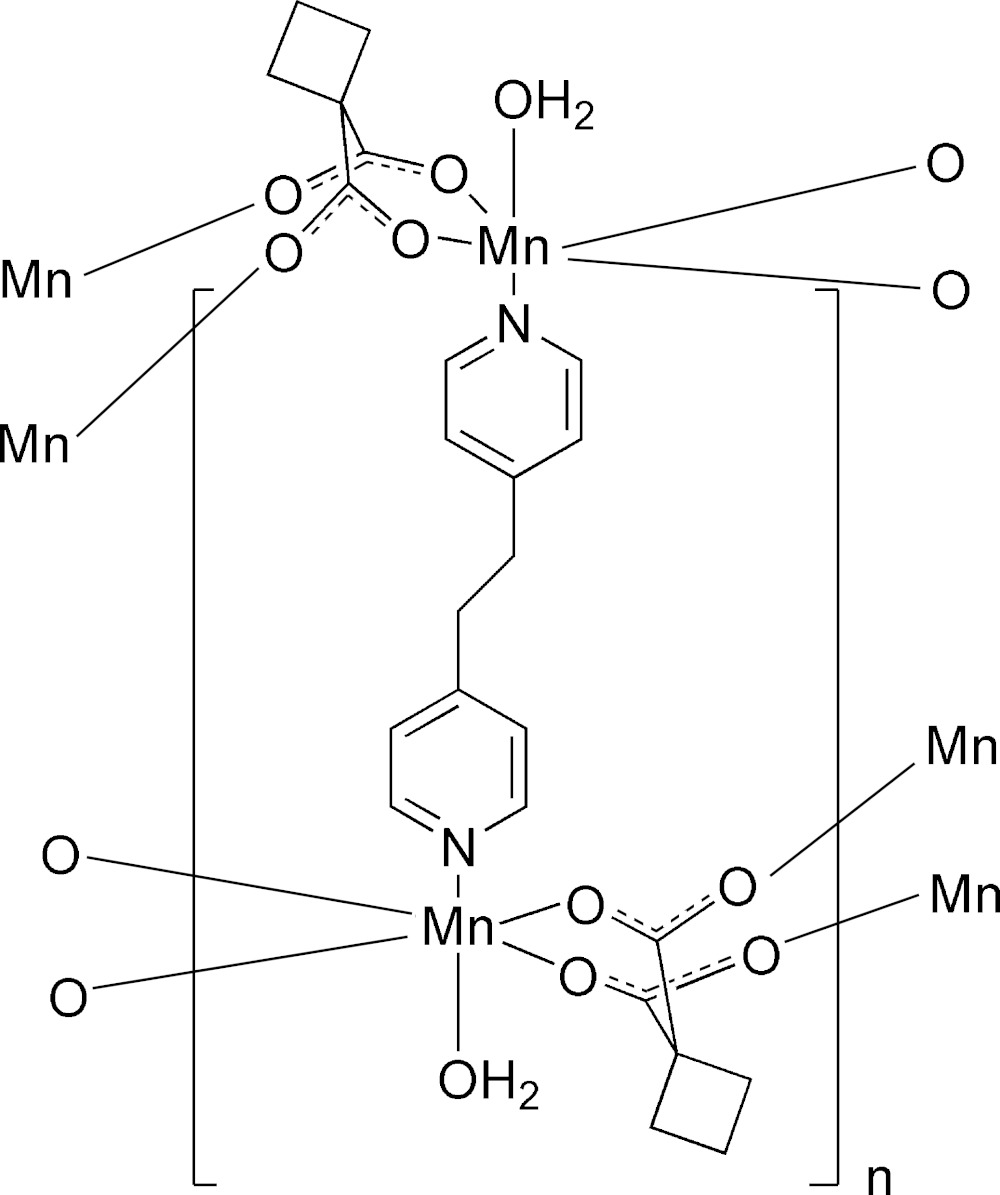


## Experimental   

### Crystal data  

[Mn(C_6_H_6_O_4_)(C_12_H_12_N_2_)(H_2_O)]
*M* *_r_* = 307.18
Monoclinic, 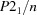

*a* = 7.4300 (15) Å
*b* = 24.095 (5) Å
*c* = 7.5930 (15) Å
β = 91.27 (3)°
*V* = 1359.0 (5) Å^3^
*Z* = 4
Mo *K*α radiation
μ = 0.99 mm^−1^
*T* = 293 K
0.13 × 0.08 × 0.05 mm

### Data collection  

Bruker APEX CCD diffractometer
Absorption correction: multi-scan (*SADABS*; Bruker, 1997 ▸) *T*_min_ = 0.88, *T*_max_ = 0.95
7527 measured reflections
2662 independent reflections
2125 reflections with *I* > 2σ(*I*)
*R*_int_ = 0.034

### Refinement  

*R*[*F* ^2^ > 2σ(*F* ^2^)] = 0.037
*wR*(*F* ^2^) = 0.097
*S* = 1.06
2662 reflections
178 parameters
2 restraints
H atoms treated by a mixture of independent and constrained refinement
Δρ_max_ = 0.39 e Å^−3^
Δρ_min_ = −0.29 e Å^−3^

### 

Data collection: *SMART* (Bruker, 1997 ▸); cell refinement: *SAINT* (Bruker, 1997 ▸); data reduction: *SAINT*; program(s) used to solve structure: *SHELXS97* (Sheldrick, 2008 ▸); program(s) used to refine structure: *SHELXL2013* (Sheldrick, 2015 ▸); molecular graphics: *SHELXTL* (Sheldrick, 2008 ▸); software used to prepare material for publication: *SHELXTL*.

## Supplementary Material

Crystal structure: contains datablock(s) global, I. DOI: 10.1107/S2056989015013791/im2468sup1.cif

**Fig. 1.**
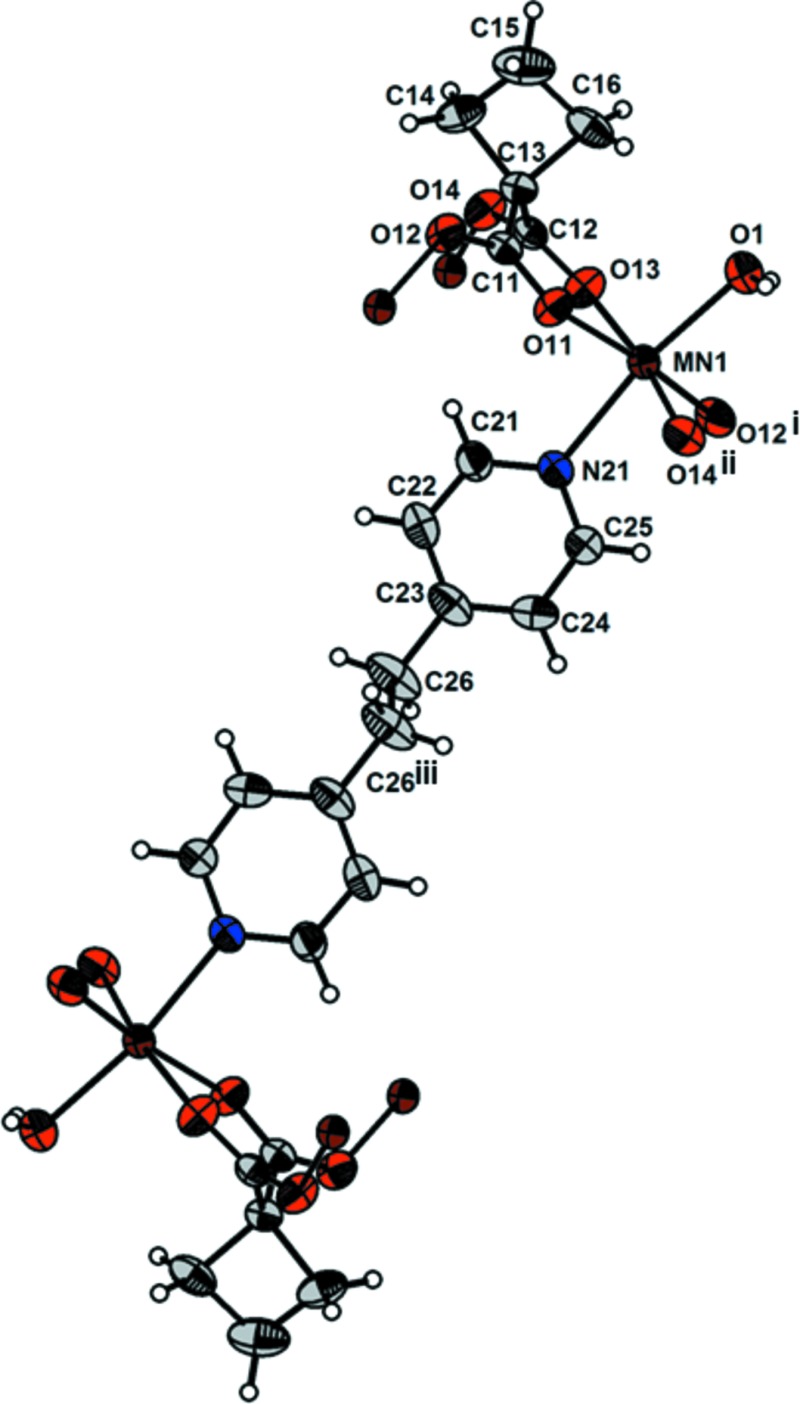
A fragment of the three-dimensional structure of the title compound showing displacement ellipsoids at the 50% probability level. Symmetry codes: (i) 1/2 + x, 1/2 - y, 1/2 + z; (ii) 1/2 + x, 1/2 - y, 1/2 + z); (iii) -x, -y, 2 - z.

Structure factors: contains datablock(s) I. DOI: 10.1107/S2056989015013791/im2468Isup2.hkl

x y z x y z x y z . DOI: 10.1107/S2056989015013791/im2468fig1.tif
A fragment of the three-dimensional structure of the title compound showing displacement ellipsoids at the 50% probability level. Symmetry codes: (i) 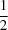 + *x*, 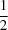 − *y*, 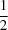 + *z*; (ii) 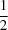 + *x*, 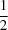 − *y*, 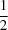 + *z*); (iii) −*x*, −*y*, 2 − *z*.

**Fig. 2.**
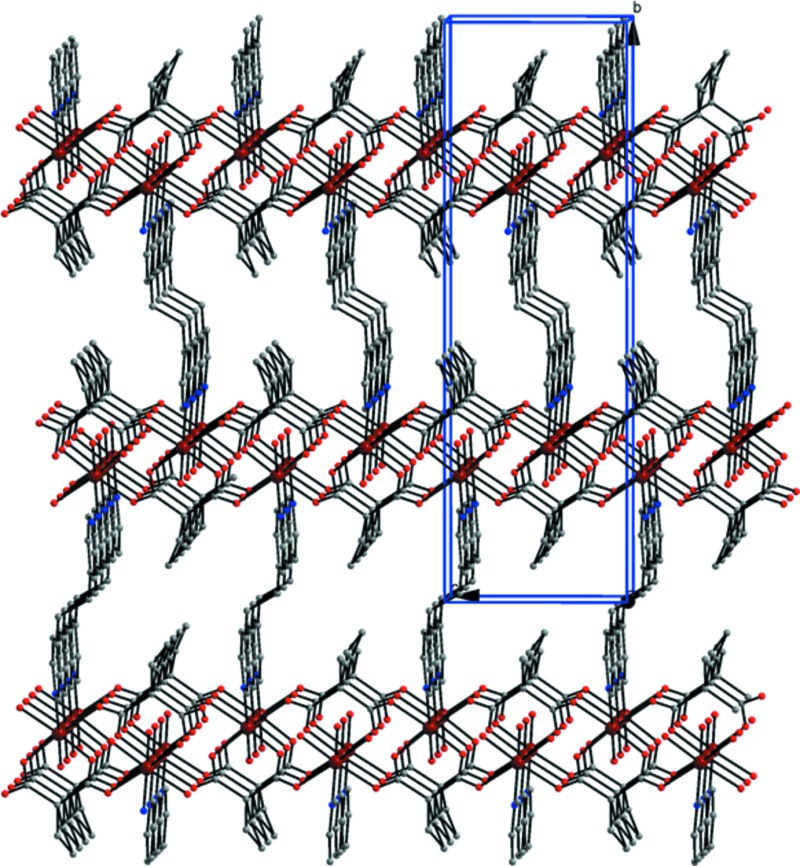
The three-dimensional framework of the title compound. All hydrogen atoms were omitted for clarity.

. DOI: 10.1107/S2056989015013791/im2468fig2.tif
The three-dimensional framework of the title compound. All hydrogen atoms were omitted for clarity.

CCDC reference: 1414117

Additional supporting information:  crystallographic information; 3D view; checkCIF report

## supplementary crystallographic information

### S1. Comment

Rigid, aromatic dicarboxylates (Sumida, *et al.*, 2012) or flexible cyclohexanedicarboxylates (Lee, *et al.*, 2011; Kim, *et al.*, 2011) have been primarily selected as the dicarboxylate ligands in coordination polymers. Flexible α,ω-alkane-dicarboxylates can also be suitable ligands for coordination polymers with different topologies. In contrast to metal complexes with aromatic dicarboxylates, few metal complexes with flexible α,ω-alkane dicarboxylates have been reported in the literature. Recently, we reported Cu-MOFs with flexible α,ω-alkane-dicarboxylate, glutarate and bipyridyl ligands (Hwang, *et al.*, 2012) and Zn-MOFs containing flexible α,ω-alkane-dicarboxylate, malonate and bipyridyl pillars (Hwang, *et al.*, 2013). Two Cu-MOFs possessed very similar pore shapes with controllable pore dimensions and exhibited good selectivity for CO_2_ over N_2_ and H_2_, and one MOF appeared to be an efficient, mild, and easily recyclable heterogeneous catalyst for the transesterification of esters (Hwang, *et al.*, 2012). A series of Zn-MOFs containing malonates and bipyridyl pillars formed three-dimensional (3-D) frameworks, and they catalyzed a heterogeneous transesterification reaction of phenyl acetate (Hwang, *et al.*, 2013). We report here on new structure of poly{[µ_2_-1,2-di(pyridin-4-yl)ethane]-bis[aqua-(µ_3_-cyclobutane-1,1-dicarboxylato)]manganese(II)}, [Mn(H_2_O)(µ_3_-C_6_H_6_O_4_)(µ_2_-C_12_H_12_N_2_)]*_n_*.

One of the repeating units of the polymeric title compound is shown in Fig. 1 and the three-dimensional packing of the title compound is presented in Fig. 2. In the title compound, [Mn(H_2_O)(µ_3_-C_6_H_6_O_4_)(µ_2_-C_12_H_12_N_2_)]*_n_*, the cbdc ligands bridge three manganese(II) ions to form two-dimensional layers. These layers are additionally connected by dipyridyl-ethane ligands to form a three-dimensional polymeric framework. The central C—C bond of the dipyridyl-ethane ligand represents a crystallographic centre of inversion. The coordination geometry of each manganese(II) ion is distorted octahedral and is built up by four carboxylate oxygen atoms, one water oxygen atom, and a pyridyl nitrogen atom. The pyridine ligand and the coordinated water molecule are in a trans-configuration. The cyclobutane-1,1-dicarboxylate ligands bridge three manganese atoms. One carboxylate unit acts as a chelating ligand towards one manganese, whereas the second carboxylate group coordinates two different manganese atoms.

### S2. Experimental

Cyclobutane-1,1-dicarboxylic acid (0.08 mmol, 11.6 mg) and Mn(NO_3_)_2_.H_2_O (0.08 mmol) were dissolved in 4 ml H_2_O and carefully layered by 4 ml of an ethanolic solution of 1,2-di(pyridin-4-yl)ethane (104.22 mg, 0.08 mmol). Suitable crystals of the title compound were obtained in a few weeks (yield: 18.5 mg, 75.3%).

### S3. Refinement

H atoms bonded to C atoms were placed in calculated positions with C—H distances of 0.93 (pyridyl) and 0.97 (cyclobutane) Å. They were included in the refinement using the riding-motion approximation with *U*_iso_(H) = 1.2 *U*_eq_(C). The positions of the H atoms of the water ligand were refined with a distance of 0.83 Å and *U*_iso_(H) = 1.2 *U*_eq_(O).

### Crystal data

**Table:**

[Mn(C_6_H_6_O_4_)(C_12_H_12_N_2_)(H_2_O)]	*Z* = 4
*M**_r_* = 307.18	*F*(000) = 632
Monoclinic, *P*2_1_/*n*	*D*_x_ = 1.501 Mg m^−^^3^
*a* = 7.4300 (15) Å	Mo *K*α radiation, λ = 0.71073 Å
*b* = 24.095 (5) Å	µ = 0.99 mm^−^^1^
*c* = 7.5930 (15) Å	*T* = 293 K
β = 91.27 (3)°	Block, colorless
*V* = 1359.0 (5) Å^3^	0.13 × 0.08 × 0.05 mm

### Data collection

**Table:**

Bruker APEX CCD diffractometer	2125 reflections with *I* > 2σ(*I*)
φ and ω scans	*R*_int_ = 0.034
Absorption correction: multi-scan (*SADABS*; Bruker, 1997)	θ_max_ = 26.0°, θ_min_ = 1.7°
*T*_min_ = 0.88, *T*_max_ = 0.95	*h* = −8→9
7527 measured reflections	*k* = −21→29
2662 independent reflections	*l* = −9→9

### Refinement

**Table:**

Refinement on *F*^2^	2 restraints
Least-squares matrix: full	Hydrogen site location: mixed
*R*[*F*^2^ > 2σ(*F*^2^)] = 0.037	H atoms treated by a mixture of independent and constrained refinement
*wR*(*F*^2^) = 0.097	*w* = 1/[σ^2^(*F*_o_^2^) + (0.0463*P*)^2^ + 0.1811*P*] where *P* = (*F*_o_^2^ + 2*F*_c_^2^)/3
*S* = 1.06	(Δ/σ)_max_ = 0.001
2662 reflections	Δρ_max_ = 0.39 e Å^−^^3^
178 parameters	Δρ_min_ = −0.29 e Å^−^^3^

### Special details

**Table:**

Geometry. All e.s.d.'s (except the e.s.d. in the dihedral angle between two l.s. planes) are estimated using the full covariance matrix. The cell e.s.d.'s are taken into account individually in the estimation of e.s.d.'s in distances, angles and torsion angles; correlations between e.s.d.'s in cell parameters are only used when they are defined by crystal symmetry. An approximate (isotropic) treatment of cell e.s.d.'s is used for estimating e.s.d.'s involving l.s. planes.

### Fractional atomic coordinates and isotropic or equivalent isotropic displacement parameters (Å^2^)

**Table:**

	*x*	*y*	*z*	*U*_iso_*/*U*_eq_	
Mn1	0.56903 (5)	0.22314 (2)	0.89895 (4)	0.02572 (14)	
O1	0.7988 (3)	0.28188 (7)	0.9016 (2)	0.0376 (4)	
H1A	0.862 (3)	0.2740 (10)	0.990 (2)	0.045*	
H1B	0.845 (4)	0.2733 (10)	0.8068 (19)	0.045*	
O11	0.4039 (2)	0.26636 (7)	1.0848 (2)	0.0349 (4)	
O12	0.1878 (2)	0.32060 (7)	1.1812 (2)	0.0391 (4)	
O13	0.4141 (2)	0.27030 (7)	0.7117 (2)	0.0365 (4)	
O14	0.1867 (2)	0.32247 (7)	0.6172 (2)	0.0384 (4)	
N21	0.3644 (3)	0.15190 (8)	0.8880 (3)	0.0340 (5)	
C11	0.2977 (3)	0.30685 (9)	1.0674 (3)	0.0275 (5)	
C12	0.3020 (3)	0.30895 (9)	0.7320 (3)	0.0269 (5)	
C13	0.3075 (3)	0.34313 (9)	0.9013 (3)	0.0288 (5)	
C14	0.1857 (4)	0.39463 (11)	0.9051 (3)	0.0446 (7)	
H14A	0.1467	0.4074	0.7893	0.053*	
H14B	0.0844	0.391	0.9826	0.053*	
C15	0.3422 (5)	0.42786 (12)	0.9847 (4)	0.0629 (9)	
H15A	0.3388	0.4313	1.1118	0.075*	
H15B	0.3588	0.4638	0.9299	0.075*	
C16	0.4729 (4)	0.38341 (11)	0.9212 (3)	0.0437 (7)	
H16A	0.5613	0.3719	1.0097	0.052*	
H16B	0.5294	0.3923	0.8109	0.052*	
C21	0.1888 (4)	0.15938 (11)	0.9101 (3)	0.0403 (6)	
H21	0.1467	0.1955	0.9207	0.048*	
C22	0.0664 (4)	0.11689 (12)	0.9181 (3)	0.0440 (7)	
H22	−0.0548	0.1246	0.9338	0.053*	
C23	0.1237 (4)	0.06274 (12)	0.9029 (4)	0.0469 (7)	
C24	0.3048 (4)	0.05483 (12)	0.8781 (5)	0.0596 (9)	
H24	0.35	0.0191	0.8663	0.072*	
C25	0.4190 (4)	0.09962 (12)	0.8707 (4)	0.0535 (8)	
H25	0.5406	0.093	0.8527	0.064*	
C26	−0.0063 (5)	0.01499 (14)	0.9168 (4)	0.0657 (10)	
H26A	−0.1279	0.0291	0.9013	0.079*	
H26B	0.0152	−0.0108	0.8215	0.079*	

### Atomic displacement parameters (Å^2^)

**Table:**

	*U*^11^	*U*^22^	*U*^33^	*U*^12^	*U*^13^	*U*^23^
Mn1	0.0270 (2)	0.0301 (2)	0.0201 (2)	−0.00067 (14)	0.00202 (14)	0.00066 (14)
O1	0.0372 (11)	0.0463 (11)	0.0294 (10)	−0.0050 (8)	0.0007 (8)	0.0017 (9)
O11	0.0431 (11)	0.0388 (10)	0.0232 (9)	0.0110 (8)	0.0089 (8)	0.0047 (7)
O12	0.0472 (11)	0.0397 (10)	0.0310 (9)	0.0081 (8)	0.0183 (8)	0.0036 (8)
O13	0.0450 (11)	0.0422 (11)	0.0219 (8)	0.0107 (8)	−0.0043 (7)	−0.0054 (7)
O14	0.0417 (10)	0.0422 (10)	0.0308 (9)	0.0082 (8)	−0.0114 (8)	−0.0067 (8)
N21	0.0326 (12)	0.0349 (12)	0.0344 (12)	−0.0048 (9)	0.0026 (9)	−0.0005 (9)
C11	0.0328 (13)	0.0282 (13)	0.0215 (11)	−0.0059 (10)	0.0009 (10)	−0.0027 (9)
C12	0.0314 (13)	0.0286 (13)	0.0208 (11)	−0.0061 (10)	0.0025 (10)	0.0018 (9)
C13	0.0371 (14)	0.0269 (13)	0.0227 (12)	−0.0027 (10)	0.0033 (10)	−0.0017 (10)
C14	0.067 (2)	0.0358 (15)	0.0306 (14)	0.0140 (14)	−0.0014 (13)	−0.0004 (11)
C15	0.103 (3)	0.0377 (17)	0.0479 (19)	−0.0099 (17)	0.0024 (18)	−0.0060 (14)
C16	0.0573 (18)	0.0441 (16)	0.0300 (14)	−0.0205 (14)	0.0047 (13)	0.0004 (12)
C21	0.0338 (14)	0.0409 (16)	0.0463 (16)	−0.0045 (12)	0.0022 (12)	−0.0035 (12)
C22	0.0344 (15)	0.0556 (18)	0.0422 (16)	−0.0112 (13)	0.0041 (12)	−0.0015 (13)
C23	0.0491 (17)	0.0491 (18)	0.0425 (16)	−0.0197 (14)	0.0015 (13)	0.0086 (13)
C24	0.060 (2)	0.0306 (16)	0.088 (3)	−0.0046 (14)	0.0037 (18)	0.0042 (15)
C25	0.0392 (16)	0.0377 (17)	0.084 (2)	−0.0003 (13)	0.0101 (16)	0.0048 (15)
C26	0.074 (2)	0.064 (2)	0.059 (2)	−0.0362 (18)	−0.0118 (18)	0.0165 (16)

### Geometric parameters (Å, º)

**Table:**

Mn1—O13	2.1369 (18)	C14—C15	1.525 (4)
Mn1—O14^i^	2.1571 (17)	C14—H14A	0.97
Mn1—O11	2.1583 (16)	C14—H14B	0.97
Mn1—O12^ii^	2.1650 (17)	C15—C16	1.531 (4)
Mn1—O1	2.2175 (19)	C15—H15A	0.97
Mn1—N21	2.293 (2)	C15—H15B	0.97
O1—H1A	0.830 (2)	C16—H16A	0.97
O1—H1B	0.830 (2)	C16—H16B	0.97
O11—C11	1.260 (3)	C21—C22	1.372 (4)
O12—C11	1.247 (3)	C21—H21	0.93
O12—Mn1^iii^	2.1650 (17)	C22—C23	1.378 (4)
O13—C12	1.261 (3)	C22—H22	0.93
O14—C12	1.252 (3)	C23—C24	1.376 (4)
O14—Mn1^iv^	2.1570 (17)	C23—C26	1.507 (4)
N21—C25	1.331 (3)	C24—C25	1.375 (4)
N21—C21	1.331 (3)	C24—H24	0.93
C11—C13	1.538 (3)	C25—H25	0.93
C12—C13	1.526 (3)	C26—C26^v^	1.457 (6)
C13—C14	1.537 (3)	C26—H26A	0.97
C13—C16	1.570 (3)	C26—H26B	0.97
			
O13—Mn1—O14^i^	170.14 (7)	C15—C14—H14A	113.8
O13—Mn1—O11	82.69 (6)	C13—C14—H14A	113.8
O14^i^—Mn1—O11	88.28 (7)	C15—C14—H14B	113.8
O13—Mn1—O12^ii^	88.48 (7)	C13—C14—H14B	113.8
O14^i^—Mn1—O12^ii^	99.99 (7)	H14A—C14—H14B	111.0
O11—Mn1—O12^ii^	168.94 (7)	C14—C15—C16	89.5 (2)
O13—Mn1—O1	94.01 (7)	C14—C15—H15A	113.7
O14^i^—Mn1—O1	91.12 (7)	C16—C15—H15A	113.7
O11—Mn1—O1	97.75 (7)	C14—C15—H15B	113.7
O12^ii^—Mn1—O1	89.47 (7)	C16—C15—H15B	113.7
O13—Mn1—N21	91.54 (7)	H15A—C15—H15B	111.0
O14^i^—Mn1—N21	84.46 (7)	C15—C16—C13	87.8 (2)
O11—Mn1—N21	89.93 (7)	C15—C16—H16A	114.0
O12^ii^—Mn1—N21	83.63 (7)	C13—C16—H16A	114.0
O1—Mn1—N21	171.03 (7)	C15—C16—H16B	114.0
Mn1—O1—H1A	106.3 (19)	C13—C16—H16B	114.0
Mn1—O1—H1B	100 (2)	H16A—C16—H16B	111.2
H1A—O1—H1B	114 (3)	N21—C21—C22	123.9 (3)
C11—O11—Mn1	131.98 (14)	N21—C21—H21	118.1
C11—O12—Mn1^iii^	133.16 (16)	C22—C21—H21	118.1
C12—O13—Mn1	131.20 (15)	C21—C22—C23	119.8 (3)
C12—O14—Mn1^iv^	131.45 (16)	C21—C22—H22	120.1
C25—N21—C21	116.3 (2)	C23—C22—H22	120.1
C25—N21—Mn1	120.58 (17)	C24—C23—C22	116.6 (2)
C21—N21—Mn1	123.01 (17)	C24—C23—C26	122.3 (3)
O12—C11—O11	123.5 (2)	C22—C23—C26	121.1 (3)
O12—C11—C13	117.4 (2)	C25—C24—C23	120.2 (3)
O11—C11—C13	119.0 (2)	C25—C24—H24	119.9
O14—C12—O13	123.4 (2)	C23—C24—H24	119.9
O14—C12—C13	116.8 (2)	N21—C25—C24	123.3 (3)
O13—C12—C13	119.7 (2)	N21—C25—H25	118.3
C12—C13—C14	116.5 (2)	C24—C25—H25	118.3
C12—C13—C11	112.53 (18)	C26^v^—C26—C23	114.2 (3)
C14—C13—C11	113.9 (2)	C26^v^—C26—H26A	108.7
C12—C13—C16	114.90 (19)	C23—C26—H26A	108.7
C14—C13—C16	87.64 (19)	C26^v^—C26—H26B	108.7
C11—C13—C16	108.9 (2)	C23—C26—H26B	108.7
C15—C14—C13	89.3 (2)	H26A—C26—H26B	107.6
			
Mn1^iii^—O12—C11—O11	−20.6 (4)	C12—C13—C14—C15	−134.6 (2)
Mn1^iii^—O12—C11—C13	161.91 (16)	C11—C13—C14—C15	91.8 (2)
Mn1—O11—C11—O12	166.53 (16)	C16—C13—C14—C15	−17.9 (2)
Mn1—O11—C11—C13	−16.1 (3)	C13—C14—C15—C16	18.3 (2)
Mn1^iv^—O14—C12—O13	22.8 (4)	C14—C15—C16—C13	−17.9 (2)
Mn1^iv^—O14—C12—C13	−159.34 (15)	C12—C13—C16—C15	136.1 (2)
Mn1—O13—C12—O14	−159.83 (17)	C14—C13—C16—C15	17.8 (2)
Mn1—O13—C12—C13	22.4 (3)	C11—C13—C16—C15	−96.7 (2)
O14—C12—C13—C14	−6.7 (3)	C25—N21—C21—C22	1.0 (4)
O13—C12—C13—C14	171.3 (2)	Mn1—N21—C21—C22	−174.6 (2)
O14—C12—C13—C11	127.5 (2)	N21—C21—C22—C23	0.0 (4)
O13—C12—C13—C11	−54.5 (3)	C21—C22—C23—C24	−0.7 (4)
O14—C12—C13—C16	−107.1 (3)	C21—C22—C23—C26	178.1 (3)
O13—C12—C13—C16	70.9 (3)	C22—C23—C24—C25	0.4 (5)
O12—C11—C13—C12	−131.6 (2)	C26—C23—C24—C25	−178.4 (3)
O11—C11—C13—C12	50.8 (3)	C21—N21—C25—C24	−1.3 (4)
O12—C11—C13—C14	3.8 (3)	Mn1—N21—C25—C24	174.4 (3)
O11—C11—C13—C14	−173.7 (2)	C23—C24—C25—N21	0.6 (5)
O12—C11—C13—C16	99.8 (2)	C24—C23—C26—C26^v^	74.0 (5)
O11—C11—C13—C16	−77.8 (3)	C22—C23—C26—C26^v^	−104.7 (5)

Symmetry codes: (i) *x*+1/2, −*y*+1/2, *z*+1/2; (ii) *x*+1/2, −*y*+1/2, *z*−1/2; (iii) *x*−1/2, −*y*+1/2, *z*+1/2; (iv) *x*−1/2, −*y*+1/2, *z*−1/2; (v) −*x*, −*y*, −*z*+2.
