# Supplementary figures and images for: Structure of poly[di­aqua­[μ-1,2-bis­(pyri­din-4-yl)ethane-κ2 N:N′]bis­(μ3-cyclo­butane-1,1-di­carboxyl­ato-κ3 O,O′:O′′:O′′′)dimanganese(II)]
Source: Acta Crystallogr E Crystallogr Commun. 2015 Jul 25;71(Pt 8):m150–1. doi: 10.1107/S2056989015013791 (PMC4571379; doi:10.1107/S2056989015013791)

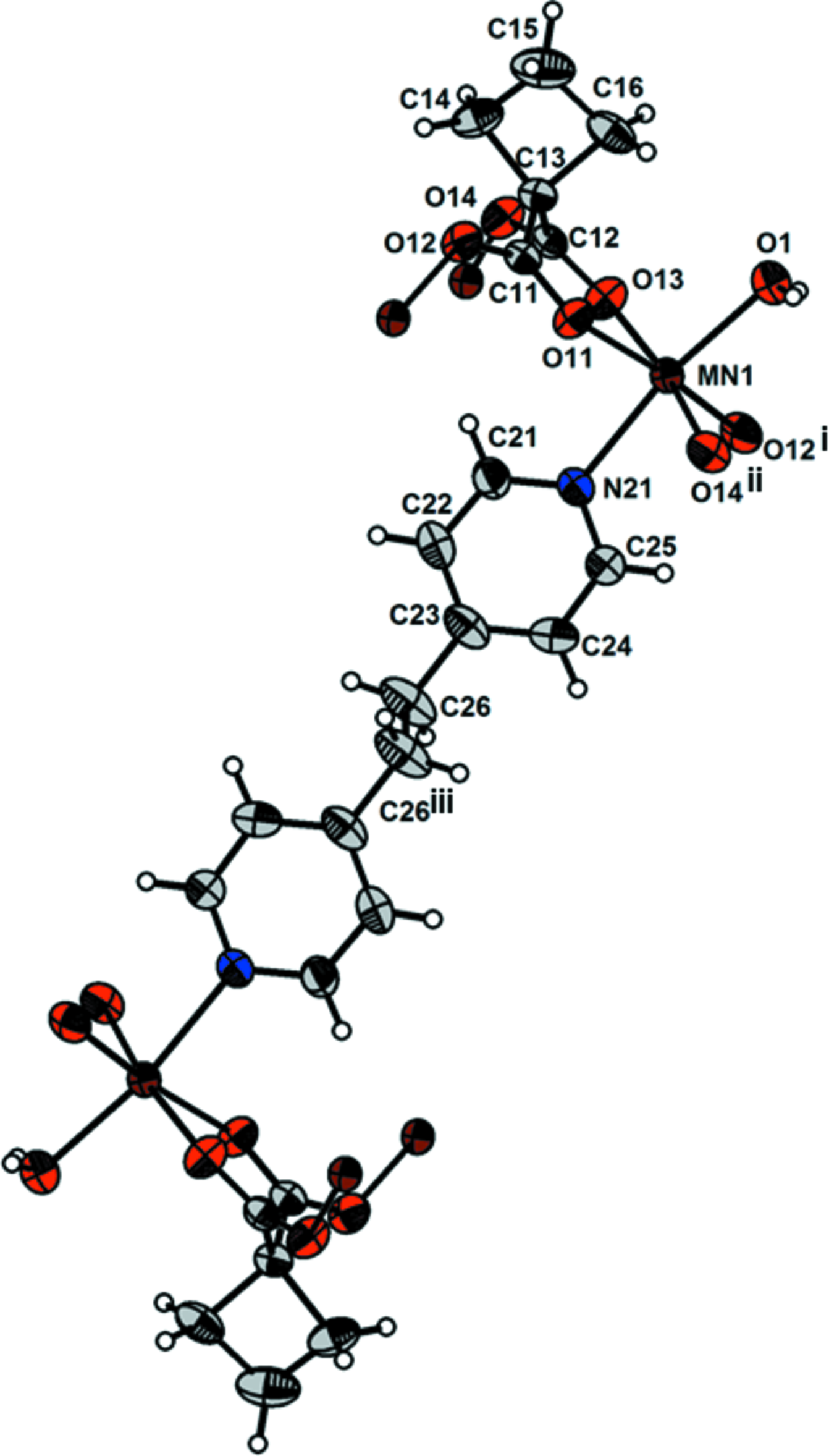

Supplement: Supplementary file 3 [file e-71-0m150-fig1.tif]

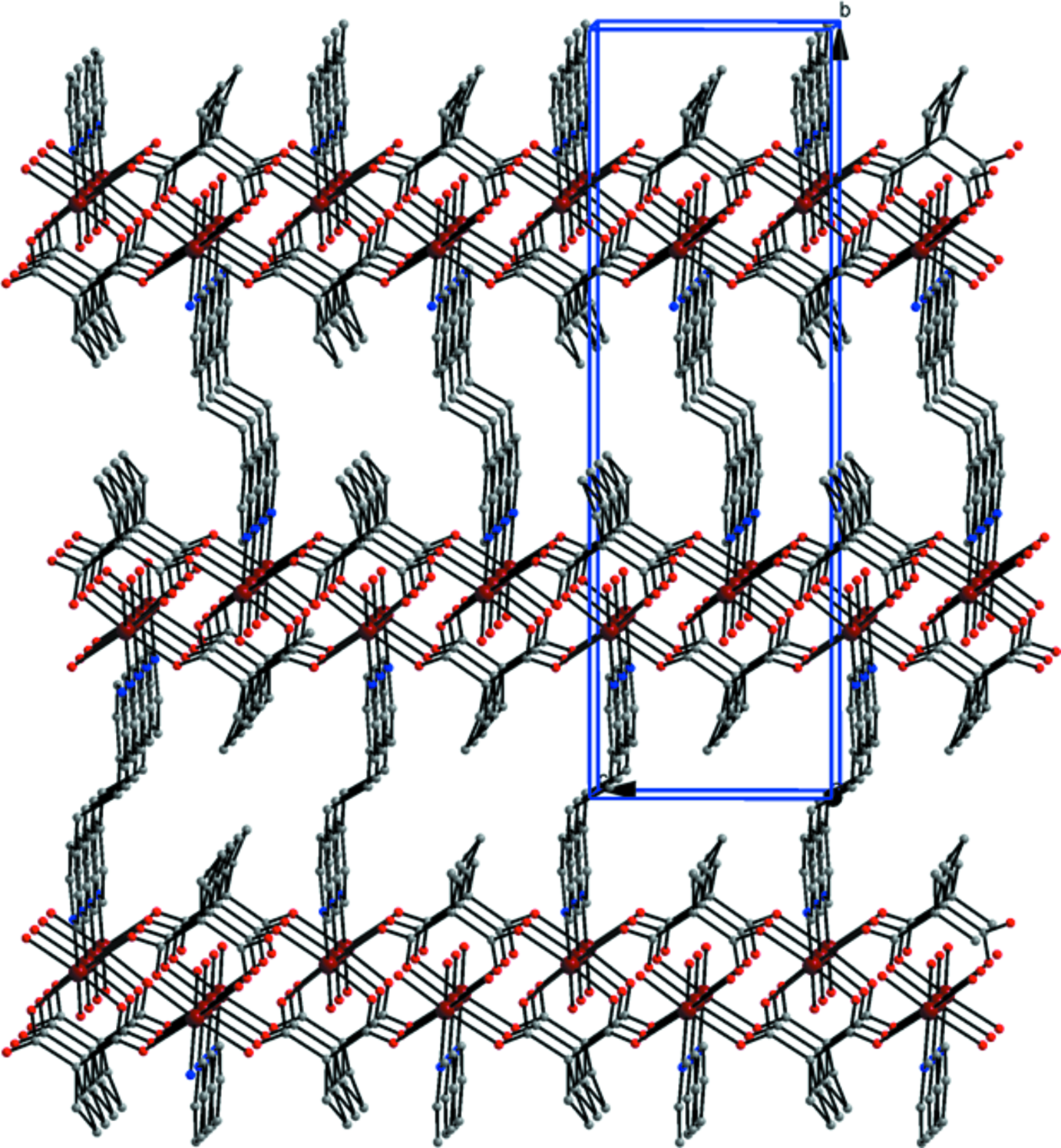

Supplement: Supplementary file 4 [file e-71-0m150-fig2.tif]
